# Effect of Laser Irradiation and Tensile Stress on Microstructure and Magnetic Properties of Fe-Based Amorphous Alloys

**DOI:** 10.3390/nano14010058

**Published:** 2023-12-25

**Authors:** Yunxia Yao, Haoxuan Huang, Cai Chen, Mayan Ni, Sen Yang

**Affiliations:** 1 Sino-French Engineer School, Nanjing University of Science and Technology, Xiaolingwei 200, Nanjing 210094, China; yunxia.yao@njust.edu.cn (Y.Y.); orange-ade@outlook.com (H.H.); cai.chen@njust.edu.cn (C.C.); ntrgnmy1117@126.com (M.N.); 2 School of Materials Science and Engineering, Nanjing University of Science and Technology, Xiaolingwei 200, Nanjing 210094, China

**Keywords:** Fe-based nanocrystalline, laser irradiation, tensile stress, soft magnetic properties

## Abstract

The effect of laser irradiation and tensile stress on the microstructure and soft magnetic properties of the FeSiBNbCu nanocrystalline alloy prepared using a continuous laser has been investigated. The experimental results indicate that a decreased laser scanning speed provides more thermal energy to induce nanocrystals and encourage grain growth. When the scanning speed is excessively high, the crystallization process will cease due to a lack of energy to drive diffusion phase transitions. Nevertheless, the introduction of tensile stress could significantly promote crystallization in FeSiBNbCu alloy samples irradiated at these high laser scanning speeds. This phenomenon can be attributed to the augmentation of compressive thermal stress at the interface between the laser-treated track and the untreated region. This heightened compressive stress promotes the diffusivity of atoms, and, as a result, the transformation from amorphous to crystalline states can be enhanced. As the applied tensile stress increases, both grain size and crystalline volume fraction exhibit a proportional augmentation. Consequently, these changes manifest in the soft magnetic properties. The crystalline volume fraction can reach 62%, and the coercivity is 2.9 A/m at the optimized scanning speed; these values correspond to 54% and 3.3 A/m under specific tensile stress loading.

## 1. Introduction

FeSiBNbCu nanocrystalline soft magnetic alloys were first introduced by Yoshizawa et al. [1]. These alloys have been commonly known to exhibit excellent soft magnetic properties [1,2,3,4], which illustrates the advantages of their application in transformers and electrical reactors or chokes [5]. Generally, these alloys are partially devitrified through isothermal annealing in a furnace for at least 30 min at temperatures ranging from 500 to 600 °C, which are superior to the primary crystallization temperature. Soft magnetic properties are closely related to the annealing process. When the induced grain size reaches the optimal range of approximately 10–15 nm [4,6], distributed within the amorphous matrix, the structural correlation length becomes significantly smaller than the ferromagnetic domain wall width. This condition leads to an almost negligible average magneto-crystalline anisotropy [4,7], and thus yields a relatively low coercivity ($H_{c}$) and an ultrahigh initial permeability ($\mu_{i}$). Moreover, factors such as the Si partition within the nanocrystals, the crystalline volume fraction, and other microstructural attributes affected by the annealing process impact both the saturation magnetization ($M_{s}$) and the saturation magnetostriction constant [8,9], which will influence the soft magnetic properties. Therefore, precise control over the annealing process is extremely important in order to obtain superior soft magnetic properties. Previous reports have demonstrated that the crystallization of amorphous ribbons can occur at higher temperatures and shorter durations through various rapid annealing methods, such as Joule heating [10,11], microwave induction heating [12], and laser irradiation-induced heating [13,14,15,16]. Considering these reported results, laser irradiation can be an effective method to produce nanocrystals within an amorphous matrix due to its ultra-high heating rate and cooling rate, as well as its precise control over the processing parameters. However, it is important to acknowledge that differences in operating mechanisms and heating rates among distinct laser systems can yield different outcomes. Recently, researchers have also explored the production of magnetic materials using additive manufacturing directly with metal powders. This approach holds appeal for generating novel-shaped components with enhanced performance and reduced processing costs [17,18,19].

Furthermore, as an almost negligible average magneto-crystalline anisotropy and a small saturation magnetostriction constant could be obtained in this material, the soft magnetic properties could be modified through an applied magnetic field [20,21,22] or external tensile stress [23,24,25]. In addition, laser irradiation is generally executed along the longitudinal axis of amorphous ribbons at a programmable velocity. It is important to note that both ends of ribbon will experience a certain tensile stress during the continuous transmission process. In the present study, we investigate the microstructure and soft magnetic properties of Fe-based amorphous ribbons both in the absence and presence of applied tensile stress using a continuous laser annealing process. The investigation utilizes a Yb-doped fiber continuous laser, and we explore the effects of phase transformations, variations in average grain size, crystalline volume fraction, coercivity, and saturation magnetization. Through this examination, we reveal the complex relationship between laser parameters, externally applied tensile stress, and resulting microstructures. These findings provide valuable insights for achieving precise control over the soft magnetic properties of the FeSiBNbCu nanocrystalline alloy.

## 2. Materials and Methods

### 2.1. Preparation

**Figure 1:**
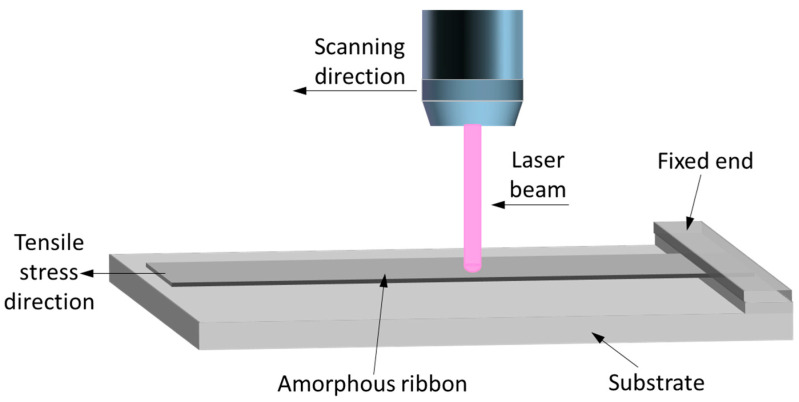
Schematic of continuous laser irradiation under external tensile stress loading.

Commercially available FeSiBCuNb amorphous ribbons (1K107B), with an average thickness of 20 μm, a width of 15 mm, and an average composition in atomic percent Fe_72_Si_16_B_7_Cu_1_Nb_4_, were used in this study; they were procured from Advanced Technology & Materials Co., Ltd. (AT&M), Beijing, China. A Yb-doped fiber continuous laser operating at a wavelength of 1064 nm and featuring a beam diameter of 3.5 mm was utilized. The amorphous ribbon sample was prepared, with a width of 15 mm and a length of 200 mm, then it was clamped at both ends with stainless steel, suspended on a stainless steel substrate, and the laser scanned along the longitudinal axis from the center of the sample. The output power was 30 W, and the scanning speed was adjusted, ranging from 7.5 mm/s to 17.5 mm/s during laser thermal treatment, without applied tensile stress. In scenarios where tensile stress was applied to one longitudinal end of the ribbon, the other end was fixed, as shown in Figure 1, and the scanning speed was maintained at 22.5 mm/s, while the stress level was altered within a range of 0 MPa to 400 MPa.

In order to compare the difference between conventional isothermal furnace annealing and laser annealing under the same raw materials, the furnace-annealed sample was prepared through annealing in a vacuum furnace under argon protection at 540 °C for 1 h.

### 2.2. Characterization

Magnetization measurements were performed using a PPMS DynaCool-9 (Quantum Design, San Diego, CA, USA.) with a maximum field of 39,800 A/m (500 Oe), and samples were prepared in ribbon form with a width of 2 mm and a length of 5 mm. Due to the fact that the coercivity of nanocrystalline soft magnetic materials is typically in the range of a few A/m, and that PPMS tests given are conducted in Oe units, the accuracy may be insufficient. Therefore, it is not suitable to use PPMS for testing the coercivity of such materials. Coercivity was then measured by a soft magnetic DC/AC testing platform, Riken Denshi BHS-40DAC, and samples were prepared in ribbon form with a width of 5 mm and a length of 60 mm. Microstructures were analyzed by a transmission electron microscopy (TEM) using a Tecnai G2 F30 S-TWIN (FEI Company, Hillsboro, OR, USA.). The crystallization characteristics of specimens were studied by X-ray diffractometer (XRD) using a Bruker D8 Advance, a Cu Kα (*λ* = 1.5406 Å) radiation source, and a scanning speed of 2.4 °/min, in a 2*θ* range from 20° to 90°. As the grain size of nanocrystal in nanocrystalline soft magnetic materials is less than 100 nm, the average grain size could be determined with a Debye–Scherrer equation by analyzing the XRD patterns [26,27], as in Equation (1):

(1) $$D=\frac{K\lambda }{\beta cos\theta }$$

where D is the average grain size, λ is the wavelength of X-ray, β is the full width at half maximum of diffraction peak, θ is the diffraction angle, and K is the Scherrer constant, ranging from about 0.62 to 2.08. The value of K is related to the reflection form and crystal form, which is usually taken as 0.89. The peaks at 65.8° were considered to determine the average grain size in this study. Additionally, the crystalline fraction volume was achieved based on the diffraction pattern area of the crystalline phase and the amorphous phase.

## 3. Results

### 3.1. Structural Characterization

**Figure 2:**
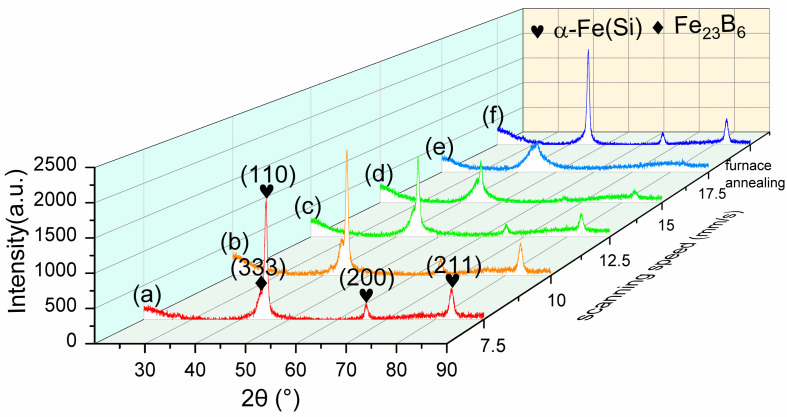
XRD patterns of laser-annealed samples with a scanning speed of (a) 7.5 mm/s, (b) 10 mm/s, (c) 12.5 mm/s, (d) 15 mm/s, (e) and 17.5 mm/s, and an isothermal furnace-annealed sample (f) annealed at 540 °C for 1 h.

Figure 2 presents the XRD pattern results under different scanning speeds, patterns (a) to (e) are the results of laser annealing samples, and pattern (f) is for the furnace-annealed sample. As the scanning speed decreases, there is a sequential enhancement in the crystallization. The onset of crystallization was observed for the sample with a scanning speed of 15 mm/s. The detected peaks correspond to α-Fe(Si) body-centered cubic (bcc) phases, specifically indexed as (110) at 45.1°, (200) at 65.8°, and (211) at 83.2°. When the scanning speed is lower than 10 mm/s, in comparison with the XRD patterns from the curve (f) in Figure 2 of isothermal furnace annealing, an additional peak attributed to Fe-B compounds appears, specifically indexed as Fe_23_B_6_ at 43.8°. Fe_23_B_6_ is a metastable face-centered cubic (bcc) phase. Fe_23_B_6_ is formed in the second stage of solidification due to the combination of Nb with Cu, this phase precipitates at the interface between the amorphous matrix and crystalline α-Fe(Si) phase [18]. 

**Figure 3:**
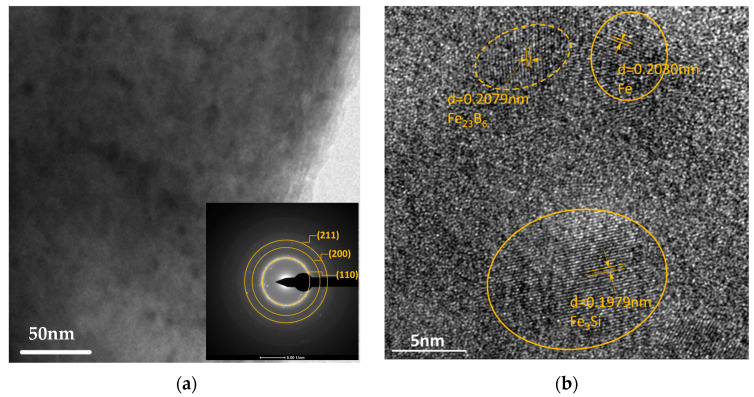
Laser-irradiated area sample with scanning speed of 10 mm/s. (**a**) Bright-field TEM and selected area diffraction (SAED) pattern and (**b**) corresponding high-resolution TEM (HRTEM) image.

Figure 3a shows the bright-field image and the corresponding selected area electron diffraction (SAED) pattern for the irradiated area in the sample with a scanning speed of 10 mm/s. The bright-field image confirms the precipitation of the nanocrystals, which distribute in the amorphous matrix. The SAED patterns show rings with intensity in all directions, indicating the precipitation of the primary α-Fe(Si) phase. The standard lattice parameters of α-Fe(Si) described in Figure 2 are 0.2027 nm, 0.1433 nm, and 0.1170 nm for crystal planes (110), (200), and (211), respectively. Additionally, the lattice parameter for Fe_23_B_6_ is 0.2071 nm. When the scanning speed changes, the concentration of Si in nanocrystals will change, which may affect the lattice parameter. The fully DO_3_ structure of crystalline Fe_3_Si (110) has a lattice parameter of 0.1990 nm. Figure 3b presents a high-resolution TEM image, and the interplanar spacing d of pure iron α-Fe (0.2030 nm) could be determined; we also found nanocrystals of Fe_3_Si (0.1979 nm), and a metastable Fe_23_B_6_ (0.2079 nm) phase was distributed in the amorphous matrix. 

**Figure 4:**
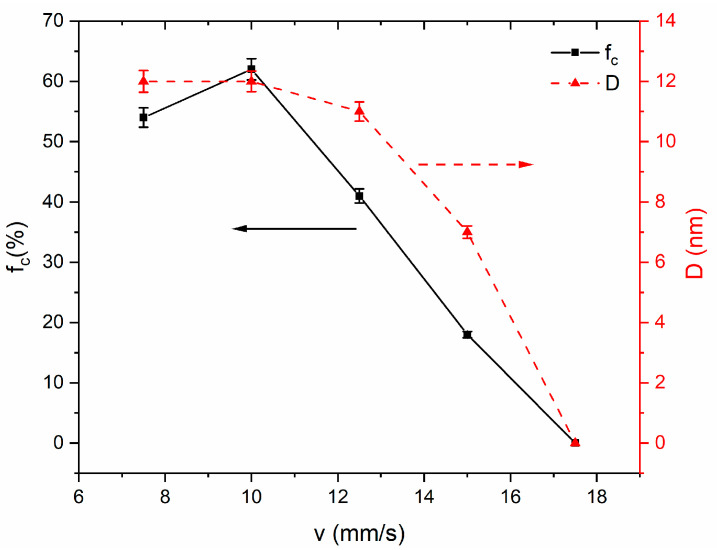
Dependence of crystalline volume fraction (f_c_) and average grain size (D) on different scanning speeds (v) with a laser output power of 30 W.

Figure 4 shows that the average grain size and crystalline volume fraction exhibit increments as the scanning speed decreases, as deduced from the analysis of XRD patterns (a) to (e) in Figure 2. When the scanning speed ranges from 15 mm/s to 7.5 mm/s, the crystallization process has been enhanced, the maximum crystalline volume fraction of 62% is achieved at 10 mm/s, and the average grain size ranges from 7 nm to 12 nm. Compared to the furnace-annealed sample (f), which represents a crystalline volume of 72%, the average grain size is approximately 10 nm. As laser annealing has an ultrahigh heating rate, the devitrification regions are limited, leading to relatively low crystalline fraction volumes. It is observed that the crystallization ceases when the scanning speed exceeds 17.5 mm/s. 

**Figure 5:**
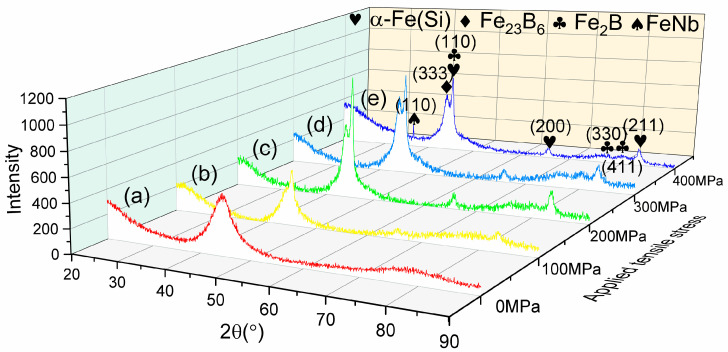
XRD patterns of laser-annealed samples with a laser power of 30 W and a scanning speed of 22.5 mm/s under tensile stress loading of (a) 0 MPa, (b) 100 MPa, (c) 200 MPa, (d) 300 MPa, and (e) 400 MPa.

Nevertheless, the nanocrystallization could be promoted through applying tensile stress during laser annealing, as shown in Figure 5, which presents the results of XRD patterns obtained under different levels of tensile stress by utilizing a laser power of 30 W and a scanning speed of 22.5 mm/s. As the scanning speed of 22.5 mm/s is superior to 17.5 mm/s, there is no crystallization. However, the increase in applied stress leads to an enhancement of the crystallization process. The patterns (a) to (e) in Figure 5 represent the crystallization characterization under applied tensile stress ranging from 0 MPa to 400 MPa. When compared to the XRD patterns in Figure 2, it can be observed that the Fe_23_B_6_ peaks are amplified with the application of stress. An additional phase of FeNb appears under a stress level of 400 MPa. In addition, the Fe_2_B phases appear around the peak of α-Fe(Si) at 83.2° for the samples with applied tensile stress of 300 MPa and 400 MPa. 

**Figure 6:**
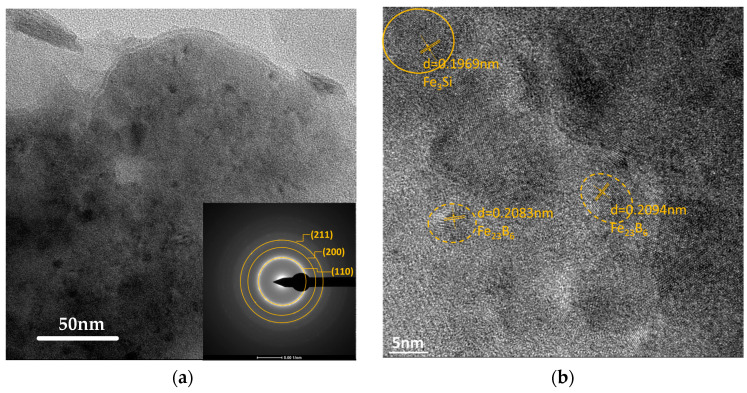
Laser-irradiated area sample with an applied stress of 300 MPa. (**a**) Bright-field TEM and selected area diffraction (SAED) pattern and (**b**) corresponding high-resolution TEM (HRTEM) image.

**Figure 7:**
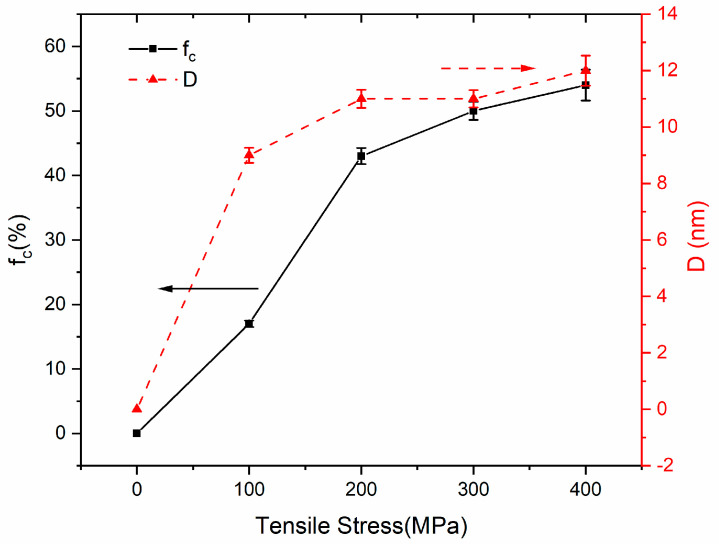
Dependence of crystalline fraction volume and average grain size on different tensile stresses.

Figure 6a shows the bright-field image, corresponding to the SAED pattern for the irradiated area in the sample with a scanning speed of 22.5 mm/s under 300 MPa tensile stress loading. The bright-field image confirms the precipitation of the nanocrystals, which distribute in the amorphous matrix. The SAED patterns show rings with intensity in all directions, indicating the precipitation of the primary α-Fe(Si) phase. Figure 6b presents a high-resolution TEM image, the interplanar spacing d of Fe_3_Si is detected, and it can also be observed that nanocrystals of the metastable Fe_23_B_6_ phase are distributed in the amorphous matrix. In comparison to the results obtained without stress, as shown in Figure 3b, Figure 6b reveals more nanocrystals in the Fe_23_B_6_ phase, which corresponds to the XRD results in Figure 5; the peaks of Fe_23_B_6_ are more evident than those without applied tensile stress. The crystalline volume fraction and average grain size were determined with the XRD patterns, as shown in Figure 7. The crystalline volume fraction and average grain size increase with increasing applied tensile stress. The maximum f_c_ reached 54%, while the D attained a maximum of 12 nm. 

### 3.2. Soft Magnetic Properties

**Figure 8:**
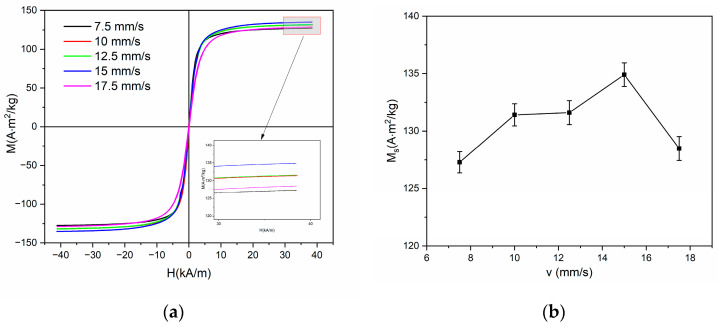
(**a**) Magnetization loops for samples at different scanning speeds and (**b**) dependence of saturation magnetization on scanning speed.

Figure 8 illustrates the performance of magnetization, Figure 8a presents the magnetization loops, and Figure 8b presents the saturation magnetization variations on scanning speed. The saturation magnetization of a sample annealed at 17.5 mm/s is approximately the same when compared to that of an unannealed ribbon by considering the uncertainty, which is 130.0 emu/g. When the scanning speed decreases to 15 mm/s, it is worth noting that an increase in saturation magnetization is observed due to the onset of crystallization [28,29]. It is also the maximum attained, which is 134.9 emu/g. Then the saturation magnetization begins to decrease with the reduction in scanning speed from 12.5 mm/s to 7.5 mm/s. The reason may correlate with the enrichment of the residual amorphous phase with Nb, which leads to a weakening of the coupling among the ferromagnetic nanograins [29]. Another factor that contributes to the decreasing saturation magnetization is the diffusion of the nonmagnetic element Si into the nanograins; the transformation from α-Fe to α-FeSi produces more non-magnetic content in nanocrystals, resulting in reduced saturation magnetization. 

**Figure 9:**
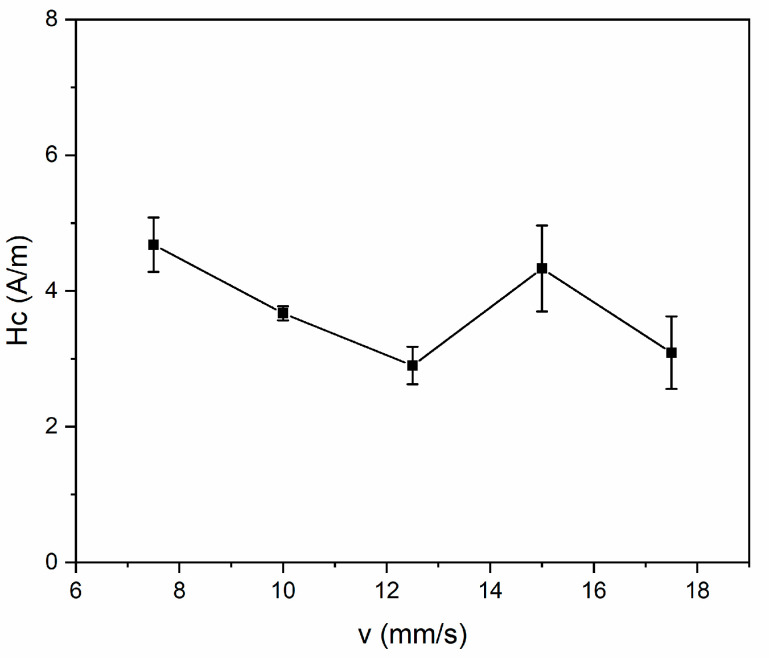
Dependence of coercivity on scanning speed.

Figure 9 illustrates the dependence of coercivity on scanning speed during laser annealing. The $H_{c}$ for the sample without annealing starts at 20.3 A/m, initially decreasing at a scanning speed of 17.5 mm/s, owing to the structural relaxation. Then, the scanning speed continue to decrease and the alloys undergo structural relaxation leading to the formation of nanocrystals. For soft magnetic nanocrystalline alloys, the excellent soft magnetic properties are closely related to the crystallization structures, which can be explained by the random anisotropy model proposed by Herzer [4]. The grain size has a strong coupling effect on the coercivity when the grain size is lower than the basic exchange length $L_{0}$; the local magneto-crystalline anisotropy is averaged as $\langle K\rangle \;and$ explained by Equation (2) [4]:

(2) $$\langle K\rangle =\beta K_{1}\cdot f_{c}^{2}{\left(D/L_{0}\right)}^{6}$$

where β is the constant to define different symmetries, $K_{1}$ is the local magneto-crystalline anisotropy, fc is the crystalline fraction volume, D is the grain size. The coercivity $H_{c}$ is directly related to $\langle K\rangle$, as presented in Equation (3) [4]:

(3) $$H_{c}=p_{c}\cdot \frac{\langle K\rangle }{J_{S}}$$

where $p_{c}$ is the dimensionless pre-factor of the order of unity and $J_{S}$ is the average saturation polarization. Then, the coercivity could be explained by Equation (4):

(4) $$H_{c}\approx p_{c}\beta K_{1}\cdot \frac{f_{c}^{2}D^{6}}{J_{S}L_{0}^{6}}$$

As the average saturation polarization relates to the saturation magnetization, the coercivity is proportional to $f_{c}^{2}$ and $D^{6}$ and is inversely proportional to the saturation magnetization. For an annealed sample ranging from 12.5 mm/s to 7.5 mm/s, the tendency of coercivity could be well explained by Equation (4). As the crystalline volume fraction and average grain size increase, and the saturation magnetization decreases, the coercivity increases with decreasing scanning speed, as shown in Figure 9. Although the coercivity performance is relatively good, the enhanced crystallization deteriorates the coercivity [30,31]. The minimum coercivity determined is 2.9 A/m at scanning speed of 12.5 mm/s, and the saturation corresponds is 131.1 emu/g. For comparison, the furnace-annealed sample in Figure 5 has a coercivity of 6.3 A/m and a saturation magnetization of 129.4 emu/g.

**Figure 10:**
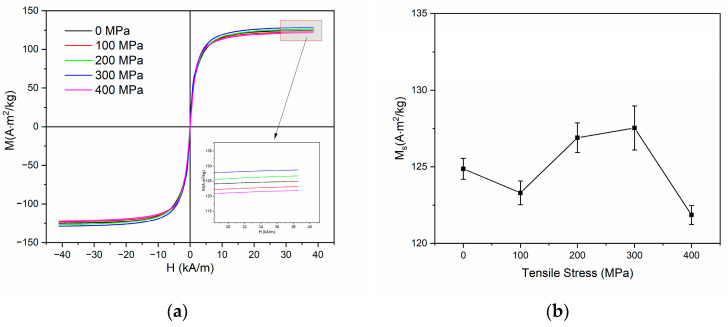
(**a**) Magnetization loops for samples at different applied tensile stress and (**b**) dependence of saturation magnetization on applied tensile stress.

Figure 10a presents the magnetization loops, and Figure 10b presents the saturation magnetization variations on different applied tensile stress. The saturation magnetization has a significant reduction with application of 400 MPa. The primary factor may be the presence of hard magnetic phases, such as Fe_2_B and FeNb, which will induce a deterioration of the magnetic softness. 

**Figure 11:**
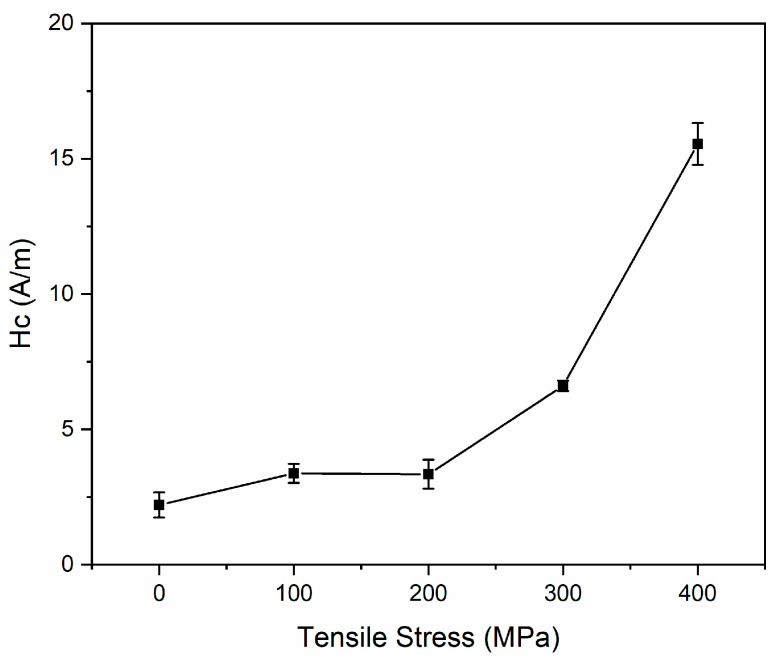
Dependence of coercivity on applied tensile stress.

Figure 11 illustrates the dependence of coercivity on applied tensile stress. The coercivity remains steady at 3.3 A/m for applied stresses of 100 MPa and 200 MPa, then it increases with increasing tensile stress due to the appearance of the hard magnetic phase Fe_2_B [8,32,33] for applied stresses of 300 MPa and 400 MPa, and the hard magnetic phase FeNb [34] for an applied stress of 400 MPa. According to Equation (4), the coercivity could be determined by the crystalline volume fraction, the average grain size, and the saturation magnetization. Although the saturation magnetization obeys firstly an increase and then a sharp decline with increasing applied tensile stress in Figure 10b, the powers of crystalline volume fraction and average grain size are greater than saturation magnetization. Therefore, the coercivity presents an incremental trend. Especially for the sample under 400 MPa loading, two different hard magnetic phases appear, the ferromagnetic coupling between grains may be destroyed, the exchange interaction between grains is then reduced, and the coercivity is subsequently not determined by domain wall movement but rather by the nucleation of magnetic domains, and the softness is seriously damaged.

## 4. Discussion

**Figure 12:**
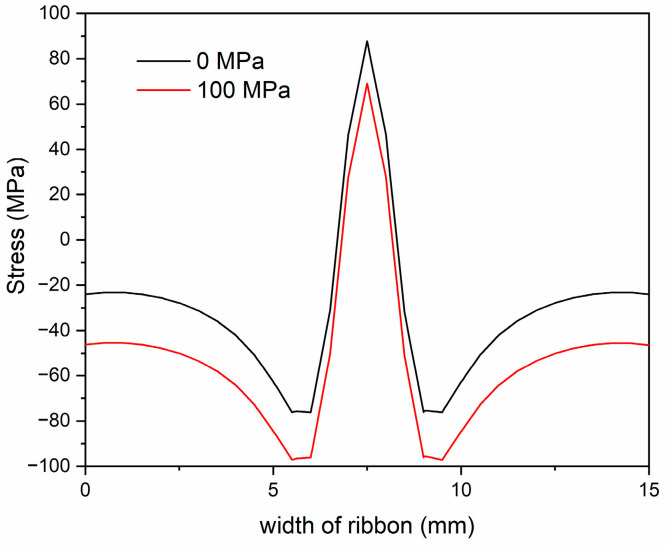
Influence of tensile stress on thermal stress across the ribbon width of the sample under a laser output power of 30 W and a scanning speed of 22.5 mm/s.

Generally, the phase transition from amorphous to nanocrystalline can be attributed to the diffusion of atoms. Diffusion in amorphous materials is believed to be induced by the stretching of bonds between adjacent atoms, thereby establishing a mechanism for atomic migration as the interstitial diffusion in crystalline materials. The relationship illustrates the dependence of the radius of nanocrystals on annealing time, and diffusivity can be explained by Equation (5) [11,35]. Assuming that the diffusivity of nanocrystal $D_{Fe(Si)}$ is independent of concentration, and nanocrystals are spherical with an average radius R, the radius of nanocrystal could be determined by:

(5) $$R=\alpha \sqrt{D_{Fe(Si)}t}$$

where *t* is the residence time, and α is a dimensionless parameter evaluated from the concentration at the nanocrystal–amorphous matrix interface [11,35]. To facilitate diffusion, an excess of free volume in the amorphous matrix plays a role in promoting atomic mobility over shorter or longer distances. This excess free volume is regarded as vacancies in crystalline materials, which determine the diffusion kinetics in amorphous materials. Consequently, the excess free volume significantly impacts the diffusion rate of atoms, and the diffusion rate within the amorphous matrix increases with an augmentation of excess free volume. For the laser irradiation without applied stress, when the scanning speed decreases, signifying an increase in residence time t, this provides sufficient laser energy density to induce nanocrystals and encourage the growth of grain. In addition, the diffusivity of nanocrystal $D_{Fe(Si)}$ is also enhanced with the increasing thermal effect induced by the decreasing laser scanning speed. Furthermore, the output of laser beam energy follows a Gaussian distribution, the thermal effect is inhomogeneous across the ribbon width, which induces a thermal stress. The thermal stress manifests as tensile stress at the center of the laser track, and contrarily the interface between the laser track and the unprocessed region experiences a compressive stress. A finite-element analysis based on the heat-transfer model using COMSOL multi-physics software (version 5.4) was developed to present these effects [36], and, applying the model to this study, the results are shown in Figure 12. Previous reports have demonstrated that the transformation from amorphous to crystalline could be promoted by a compressive stress via enhancing the diffusivity [36,37]. The compressive stress arising from localized thermal expansion in the interface facilitates the formation and propagation of shear bands. Shear bands exist as excess free volume to promote diffusion. However, when the scanning speed exceeded 17.5 mm/s, the crystallization process ceased due to insufficient activation energy. The nanocrystallization process is governed by the scanning speed.

As shown in Figure 12, the compressive stress effects are more significant in the interface when increasing the applied tensile stress. When the compressive stress effect enhances, more shear bands could be produced, which provides excess free volume to augment the diffusivity; thereby, the generation of nanocrystals may be enhanced. This phenomenon is consistent with the trend observed in grain size and crystalline volume fraction as functions of applied stress, as illustrated in Figure 5 and Figure 7.

Furthermore, compared to the previously reported nanocrystal sizes created using laser annealing, the results depicted in Figure 4 demonstrate smaller grains produced under a continuous Yb-doped fiber laser compared to those generated under a CO_2_ laser as documented by Lanotte [15]. The process of laser irradiation-induced heating is associated with rapid heating and cooling rates; thus, it will lead to nucleation dominating during the crystallization process while grain growth is restricted. Moreover, the Yb-doped fiber laser possesses a wavelength 10 times shorter than the CO_2_ laser, which means a higher heating rate and cooling rate. Higher heating and cooling rates lead to an increased nucleation density and reduced growth rates. The elevated nucleation density results in overlapping diffusion fields, impeding the growth of grains, and then resulting in a refinement of grains. However, the average grain size is slightly larger than that achieved through the furnace annealing (~10 nm) in this study. It is primarily attributable to the fact that the ultrahigh heating rate degrades the stabilization effect of Nb [12,14], Nb atoms are unable to diffuse sufficiently far away from their initial positions in short residence time [38], and results in negligibly few Nb-rich layers being formed; grain growth is predominantly restricted by both B and Fe-B compounds [12]. 

## 5. Conclusions

The evolutions of microstructures and soft magnetic properties induced by laser annealing were investigated for nanocrystalline FeSiBCuNb soft magnetic alloys. The results highlight the effects induced by laser annealing with different scanning speeds and with different applied tensile stresses. The differences between the laser annealing and the conventional furnace annealing are as follows:

(1) The crystallization process in laser annealing could be enhanced by reducing the scanning speed or increasing the tensile stress. These two methods follow distinct crystallization mechanisms, when there is no additional tensile stress, the crystallization process is determined by the thermal effect of laser irradiation and the compressive stress effect in the interface between the laser track and the unprocessed region. However, when the scanning speed is too high, it is insufficient to provide the activation energy needed to promote the transformation from amorphous to nanocrystalline. The application of an external tensile stress can ameliorate this issue, because the crystallization is promoted by the compressive stress induced at the interface between the laser track and the unprocessed region.

(2) In contrast to furnace annealing, laser annealing’s ultrahigh heating rate, the devitrification regions are limited, leading to relatively low crystalline fraction volumes. When the laser scanning speed decreases, the grain sizes vary from 7 nm to 12 nm, while the crystalline fraction volumes simultaneously increase to a maximum of 62%. Similarly, as the applied stress increases, the grain sizes increase to a maximum of 12 nm, and the crystalline volume fraction reaches at 54%. According to the soft magnetic measurements, the optimal sample treated with laser annealing exhibits a coercivity of 2.9 A/m and a saturation magnetization of 131.1 emu/g for the sample with a scanning speed of 12.5 mm/s. Additionally, the optimal sample of 22.5 mm/s with applied stress of 200 MPa possesses a coercivity of 3.3 A/m and a saturation magnetization of 126.9 emu/g, respectively.

Through further research and optimization, laser annealing represents a feasible manufacturing approach that may provide the opportunity to tailor soft magnetic performance to meet specific requirements, and thus offers significant opportunities for customization of the microstructure of amorphous and nanocrystalline alloys.

## Data Availability

The data that support the findings of this study are available within the article.
